# Cost study of metal stent placement *vs* single-dose brachytherapy in the palliative treatment of oesophageal cancer

**DOI:** 10.1038/sj.bjc.6601815

**Published:** 2004-05-04

**Authors:** S Polinder, M Y V Homs, P D Siersema, E W Steyerberg

**Affiliations:** 1 Department of Public Health, Erasmus MC/University Medical Center Rotterdam, PO Box 1738, 3000 CA Rotterdam, The Netherlands; 2 Department of Gastroenterology & Hepatology, Erasmus MC/University Medical Center Rotterdam, PO Box 2040, 3000 CA Rotterdam, The Netherlands

**Keywords:** oesophageal carcinoma, brachytherapy, self-expanding metal stent, palliation, cost analysis

## Abstract

Self-expanding metal stent placement and single-dose brachytherapy are commonly used for the palliation of oesophageal obstruction due to inoperable oesophagogastric cancer. We randomised 209 patients to the placement of an Ultraflex stent (*n*=108) or single-dose brachytherapy (12 Gy, *n*=101). Cost comparisons included comprehensive data of hospital costs, diagnostic interventions and extramural care. We acquired detailed information on health care consumption from a case record form and from monthly home visits by a specialised nurse. The initial costs of stent placement were higher than the costs of brachytherapy (€1500 *vs* €570; *P*<0.001). Total medical costs were, however, similar (stent €11 195 *vs* brachytherapy €10 078, *P*>0.20). Total hospital stay during follow-up was 11.5 days after stent placement *vs* 12.4 days after brachytherapy, which was responsible for the high intramural costs in both treatment groups (stent €6512 *vs* brachytherapy €7982, *P*>0.20). Costs for medical procedures during follow-up were higher after stent placement (stent €249 *vs* brachytherapy €168, *P*=0.002), while the costs of extramural care were similar (€1278 *vs* €1046, *P*>0.20). In conclusion, there are only small differences between the total medical costs of both palliative treatment modalities, despite the fact that the initial costs of stent placement are much higher than those of brachytherapy. Therefore, cost considerations should not play an important role in decision making on the appropriate palliative treatment strategy for patients with malignant dysphagia.

The incidence of oesophageal cancer has risen rapidly, due to a marked increase in the incidence of adenocarcinoma (Devesa *et al*, 1998; Botterweck *et al*, 2000). Oesophageal cancer is a disease with a high mortality, as reflected by a 5-year survival of 10–15% (Pisani *et al*, 1999). Moreover, more than 50% of patients with oesophageal cancer have already inoperable disease at presentation. Most of these patients require palliative treatment to relieve progressive dysphagia (Sagar *et al*, 1994). Treatment options presently available for palliation of dysphagia include self-expanding metal stent placement (Kozarek *et al*, 1996; Bartelsman *et al*, 2000; Siersema *et al*, 2001), laser therapy (Dallal *et al*, 2001), photodynamic therapy (PDT) (Luketich *et al*, 2000; Moghissi *et al*, 2000), external beam radiation in combination with brachytherapy (Taal *et al*, 1996; Schraube *et al*, 1997), brachytherapy as a sole treatment (Jager *et al*, 1995; Sur *et al*, 2002; Homs *et al*, 2003a) and dilatation (Lundell *et al*, 1989). A disadvantage of laser therapy is that repeated treatment sessions are required to achieve and maintain adequate palliation (Dallal *et al*, 2001; Spencer *et al*, 2002). A combined treatment of external beam radiation with brachytherapy is often too intensive for patients with inoperable disease due to metastases or a poor medical condition. Photodynamic therapy involves the local destruction of tumour tissue by light of a specific wavelength activating a previously administered photosensitiser, which is retained in malignant tissue. Due to the high costs of the treatment, the side effects and the necessity of repeated treatments every 6–8 weeks, PDT is not considered to be the most optimal treatment for palliation of malignant dysphagia (Lightdale *et al*, 1995). Dilatation can relieve dysphagia temporarily, but it often provides relief palliation for a short period. In many patients with inoperable disease, a stent is placed for the palliation of dysphagia. In addition, brachytherapy as a sole treatment is frequently used in Western Europe, South Africa, Japan and to a lesser extent in the USA. Both stent placement and brachytherapy have been proven to be effective in relieving dysphagia with a low complication rate, but recurrent dysphagia due to various causes is seen in 30–40% of patients (Brewster *et al*, 1995; Kozarek *et al*, 1996; Bartelsman *et al*, 2000; Siersema *et al*, 2001; Sur *et al*, 2002; Homs *et al*, 2003a).

In order to comprehensively assess the relative merits of the different palliative treatments of malignant dysphagia, health economic aspects should be incorporated. Remarkably, the economic implications of both stent placement and brachytherapy have been evaluated only in a few studies (Adam *et al*, 1997; Farndon *et al*, 1998; Nicholson *et al*, 1999; Dallal *et al*, 2001; O’Donnell *et al*, 2002). If costs were considered, these were only ‘roughly’ calculated (Dallal *et al*, 2001), using charges/fees and with little information about the differentiation of the costs. In addition, the number of patients incorporated in the studies was low (Farndon *et al*, 1998; Dallal *et al*, 2001; O’Donnell *et al*, 2002). In studies on costs, this may result in a high degree of distortion, because peaks in volumes of some expensive cost items can highly influence the average outcomes.

We aimed to study the total direct and indirect costs of brachytherapy and stent placement in the palliation of malignant dysphagia within the framework of a randomised trial. We present estimates of the full cost price, based on real resource use, in substantial patient groups.

## MATERIALS AND METHODS

### Study population

We performed a prospective study in three university hospitals and six general hospitals in the Netherlands. Between December 1999 and July 2002, 209 consecutive patients with dysphagia from inoperable carcinoma of the oesophagus or gastric cardia due to metastases and/or a poor medical condition were randomised to placement of a covered Ultraflex stent (*n*=108) or single-dose (12 Gy) brachytherapy (*n*=101). For brachytherapy, a flexible applicator (Bonvoisin-Gérard Esophageal Applicator, Nucletron, Veenendaal, The Netherlands) with a diameter of 10 mm was passed down the oesophagus. A single dose of 12 Gy was administered with the radioactive source ^192^Iridium at 1 cm from the source axis of the applicator. The study was approved by the Central Committee on Research Involving Human Subjects in the Netherlands.

### Study end points

The clinical outcomes were functional outcome, complications, persisting or recurrent dysphagia needing re-intervention, survival and quality of life, as measured by standardised questionnaires. The clinical outcomes have been presented in detail elsewhere (Homs *et al*, 2003b, 2003c). Here, we will focus on the real medical costs of the two treatment strategies. Costs were studied from a societal perspective and were estimated for the period after randomisation until death for 95% of the patients, or a follow-up of at least 9 months for the remaining 5% of patients.

### Follow-up

Patients were prospectively followed by home visits of specially trained research nurses at 14 days, 1 month and then monthly until 1 year after treatment. After 1 year, patients were visited every 3 months, and/or telephone calls to the patient and/or the patients’ general practitioner were made. For each patient, we registered the number of inpatient days, the time needed for nursing care and therapy as well as the visits to physicians and other health practitioners by a checklist filled in by the research nurse. The response was more than 90% during the entire follow-up period.

The participating clinicians filled out standardised case record forms (CRFs) during control visits, re-treatments and admissions.

### Cost calculations

Real medical costs were calculated by multiplying the volumes of health care use with the corresponding unit prices. We made a distinction between the full cost price of the interventions of brachytherapy and stent placement by itself and the total medical costs per patient during follow-up.

The calculation of the full cost price of brachytherapy and stent placement consisted of detailed measurement of investments in manpower, equipment, materials, housing and overhead. The salary schemes of hospitals and other health care suppliers were used to estimate costs per hour for each caregiver. Taxes, social securities and vacations were included, as well as the costs of the time that could not be assigned to other patients. The costs of equipment included those of depreciation, interest and maintenance.

For the calculation of the total medical costs per patient, we distinguished intramural medical costs (inpatient days, health practitioner activities, the full cost price of the medical treatment and other medical procedures) and extramural medical costs (home care, general practitioner). Costs caused by loss of production due to absence from work were not taken into account, because the majority of patients were retired from work.

For the most important cost items, unit prices were determined by following the micro-costing method (Gold *et al*, 1996), which is based on a detailed inventory and measurement of all resources used. For instance, we registered the time investments of health practitioners per patient (during the intervention). Costs for inpatient days in hospital were estimated as real, basic costs per day using detailed information from the financial department of the hospital. We made a distinction between the costs of general and university hospitals. These estimates included overhead and indirect costs. From a differential point of view, that is, comparison of the two treatment strategies, some diagnostic interventions were decided to be less relevant. We chose not to invest much time and effort in exploring costs that were unlikely to make any difference to the study result (Drummond *et al*, 1997), for example, because they were low in price or volume. For these items, we used charges as a proxy of real costs. In the Netherlands, a detailed ‘fee for service’ system is used for the remuneration of medical interventions and diagnostic procedures. In order to calculate the costs for medication, we used average charges for analgesics, antibiotics and additional medications.

Table A1 (Appendix A) gives an overview of the cost categories and data used in the cost calculations. We reported the costs in Euro for the year 2002, when 1 Euro equalised approximately 1 US dollar. Discounting was not relevant because of the limited time horizon (median survival 4–5 months).

### Statistical analysis

All analyses were performed on an intention-to-treat basis. The cost differences between brachytherapy and stent placement were analysed using the Mann–Whitney *U* test. Since cost data per patient (but not per day care) are typically highly skewed, we used nonparametric bootstrap techniques to derive a 95% confidence interval for the differences in distributions of the direct medical costs.

In a sensitivity analysis, the effect of excluding ‘palliation-related costs’ was assessed by leaving these costs out of consideration. We performed calculations assuming that nursing home admissions and nursing care at home were not directly related to both treatments, but could be attributed to the advanced stage of the disease.

## RESULTS

### Patient characteristics and clinical outcomes

The two patient groups were comparable with respect to patient and tumour characteristics. Both treatment groups consisted predominantly of males, with a mean age of 69 years (Table 1

**Table 1:** Characteristics of 209 patients randomised to brachytherapy or stent placement for palliation of malignant dysphagia

	**Brachytherapy**	**Stent placement**
Randomised	101	108
Age	69±13	69±11
Gender (male/female)	76/25	86/22
WHO performance score before treatment (mean±s.d.)	1.0±0.4	0.9±0.5
		
*Indications for treatment*		
Metastases	66 (65%)	68 (63%)
Poor medical condition	23 (23%)	28 (26%)
Both	12 (12%)	12 (11%)
		
Tumour length (cm) (mean±s.d.)	7.5±2.6	7.5±2.8
Received assigned intervention	96 (brachytherapy)	105 (stent)
Re-intervention	45 (stent) 3 (second brachytherapy)	2 (brachytherapy) 24 (second stent)
Median survival (days)	155 (95% CI: 128–182)	145 (95% CI: 104–186)
Total complications	23 in 21 patients (21%)	45 in 36 patients (33%)
Major complications	14 in 13 patients (13%)	28 in 27 patients (25%)
Minor complications	8 in 8 patients (8%)	16 in 16 patients (15%)
Persistent/recurrent dysphagia	53 in 43 patients (43%)	52 in 43 patients (40%)

).

Dysphagia improved more rapidly after stent placement than after brachytherapy. However, the overall improvement of dysphagia was better after brachytherapy. More complications occurred after stent placement (33% total complications *vs* 21% after brachytherapy; *P*=0.02). Major complications within 7 days after treatment included perforation (*n*=3), fever (*n*=2), severe pain (*n*=2) and aspiration pneumonia (*n*=2). Late major complications consisted predominantly of haemorrhage (*n*=19) occurring more frequently after stent placement (14 *vs* 5 after brachytherapy). The need for re-intervention for persistent or recurrent dysphagia was not significantly different for both groups (40 *vs* 43%, respectively). Recurrent dysphagia after stent placement was predominantly caused by tumour overgrowth (*n*=16), stent migration (*n*=18) or food bolus obstruction (*n*=16), and was treated by placement of a second stent, endoscopic stent clearance or a variety of other treatments. The majority of re-interventions after brachytherapy were caused by tumour persistence (*n*=18) or tumour recurrence (*n*=26), both most frequently treated with placement of a stent.

The median survival was similar for both treatment groups (stent 145 *vs* brachytherapy 155 days). There was an overall long-term benefit in general (EORTC QLQ C-30 and EuroQol-5D) and disease-specific quality of life scores (EORTC OES-23) in the brachytherapy group during follow-up.

### Costs

The initial cost price of treatment, based on real resource use, was much higher for stent placement (€1500) than for brachytherapy (€570). The main cause for this difference was the high purchase costs of the Ultraflex stent (€1100) (Table 2

**Table 2:** Full cost price (€, 2002) of brachytherapy and stent placement

**Cost category**	**Brachytherapy**	**Stent placement**
Personnel	152	74
Equipment	75	40
Materials	14	1307
Housing/overhead	70	40
Diagnostics	259	37
		
Total costs	570	1500

).

Table 3

**Table 3:** Average health care use and costs (€, 2002) per patient for stent placement and brachytherapy

	**Brachytherapy (*n*=101)**			**Stent placement (*n*=108)**		**95% CI^a^**
**Cost category**	**Cost price**	**Volume**	**Costs**	**Volume**	**Costs**	**Mann–Whitney**
*Treatment*						
Brachytherapy	570	0.96	547	0,019	11	
Stent placement	1500	0.58	870	1,29	1935	
			1417		1946	[253, 822] *P*<0.001
						
*Intramural care*						
Hospital (academic)	520	7.7	4006	6.9	3587	
Hospital (general)	381	4.7	1788	4.6	1760	
ICU	1450	0.02	31	0.06	72	
Nursing home	173	11	1898	4.8	838	
Physician (academic)	135	1.41	190	1.30	176	
Physician (general)	98	0.70	69	0,82	79	
			7982		6512	[–4341, 1104] *P*>0.20
						
*Medical procedures*						
Endoscopy	125	0.74	93	1.17	146	
PEG	100	0.04	4	0.04	4	
X-ray thorax	37	0.78	29	1.41	52	
X-ray abdomen	37	0.07	3	0.21	5	
Ultra-sound abdomen	42	0.05	3	0.04	2	
X-ray oesophagus	37	0.13	5	0.19	7	
ERT	39	0,81	32	0,57	22	
Adjustment ERT	98	0,15	15	0,09	9	
			168		249	[3, 137] *P*=0.002
						
*Extramural care*						
General practitioner (inpatient)	19	0,9	18	1,06	20	
General practitioner (home visit)	37	9	333	8,1	298	
Nursing care at home (specialised)	55,6	13,4	750	11,4	638	
Nursing care at home	32,4	1,9	61	2	64	
Drip-feed	8,5	13,7	116	3,05	26	
			1278		1046	[–783, 227] *P*>0.20
						
*Medication*			350		325	
						
*Total costs per patient*			11 195		10 078	[–3790, 1806] *P*>0.20
						

a Derived from 2000 bootstrap samples drawn with replacement.

gives an overview of the average health care use and costs per patient for stent placement and brachytherapy. Patients randomised to brachytherapy were admitted on average 7.1 days longer in a health care institution than patients randomised to stent placement (23.4 *vs* 16.3 days). The main reason for this was the longer period patients randomised to brachytherapy were admitted to nursing homes (11.0 *vs* 4.6 days). The average time spent in hospital was similar for both treatments (12.4 for brachytherapy and 11.5 for stent placement). The costs for intramural care were by far the highest cost category for both treatments, but differences were not statistically significant (stent placement €6512 *vs* brachytherapy €7982, *P*>0.20). Costs of medical procedures during follow-up were significantly higher for stent placement (€249) than for brachytherapy (€168) (*P*=0.002), since major complications and re-interventions occurred more often after stent placement than brachytherapy. The average costs for extramural care were €1278 for brachytherapy and €1046 for stent placement. For both treatments, this could largely be attributed to home visits by the general practitioner and specialised nursing care at home. The costs for medications were similar for brachytherapy and stent placement (€350 and €325).

The total average costs per patient for both treatments were similar at €11 195 for brachytherapy and €10 078 for stent placement (*P*>0.20). If the ‘palliation-related’ health care was not taken into consideration, then the costs of intramural and extramural care for brachytherapy and stent placement decreased. This resulted in total medical costs for brachytherapy of €8490 and for stent placement of €8538 (*P*>0.20).

## DISCUSSION

We found only small differences between the total medical costs of single-dose brachytherapy as compared to metal stent placement for the palliation of dysphagia from inoperable oesophageal carcinoma. Stent placement was initially more expensive than brachytherapy, due to the high purchase costs of the stent, but at the long term costs were comparable.

Many patients in both treatment groups needed re-intervention for persistent or recurrent dysphagia. Of the patients randomised to brachytherapy, 45 out of 101 (45%) subsequently received a stent, while 24 out of 108 (22%) of the patients randomised to stent placement received a second stent during follow-up. Since our analysis was based on the intention-to-treat principle, costs of non-assigned treatment were accounted to the randomised treatment group. Total treatment costs, which included the average costs of additional treatment plus re-intervention, were higher for stent placement. However, if the intramural and extramural health care costs were also taken into account, then these high initial costs were only a small part of the total medical costs, which resulted in similar total medical costs.

Cost comparisons between medical treatments are often based only on the initial costs. This would imply that stent placement, with the high purchase costs of the device, would be less attractive than brachytherapy. In this study, we clearly demonstrated that the total medical costs of stent placement and brachytherapy were similar when the full follow-up period was considered. This illustrates that cost comparisons between interventions may vary substantially, depending on which, and how many, components are included in a total cost equation (Sahai, 2000).

Few studies have been published on costs of brachytherapy or stent placement in the palliative treatment of oesophageal cancer. Three studies compared stent placement with plastic endoprostheses (O’Donnell *et al*, 2002), conventional therapy (Nicholson *et al*, 1999) or thermal ablative therapy (Dallal *et al*, 2001). These three studies reported corresponding initial costs for stent placement, but found lower total medical costs, compared to our study. Dallal *et al* (2001) included only the costs of the intervention and hospital stay in the total costs for stent placement (€4920), which can explain the difference in total costs compared to our study. They found a median hospital stay of 12 days after stent placement, similar to findings in our study (Table 3). Farndon *et al* (1998) compared the placement of a plastic endoprosthesis with single-dose brachytherapy and showed that the total costs of brachytherapy (€2603) were lower compared to stenting (€3564). Presently, plastic endoprostheses are no longer considered adequate for palliation of malignant dysphagia due to a high procedure-related complication rate with plastic endoprostheses (Siersema *et al*, 1998). Since there is no detailed information available on the costs in the above-mentioned articles (Farndon *et al*, 1998; Nicholson *et al*, 1999; Dallal *et al*, 2001; O’Donnell *et al*, 2002), it is not possible to explain the differences in total costs between these studies and ours. It could well be that both intramural and extramural health care use was under-reported. Finally, the number of patients included and receiving stent placement or brachytherapy was relatively low (*n*<35) in these studies (Farndon *et al*, 1998; Nicholson *et al*, 1999; Dallal *et al*, 2001; O’Donnell *et al*, 2002).

A common problem when using clinical trials for any kind of cost assessment arises from the fact that the clinical protocol mandates more visits, consultations and examinations than otherwise used in clinical practice (Myrvold *et al*, 2001). For a treatment in a research setting, there will be more costs, compared to daily practice. Therefore, we excluded protocol-driven medical care such as visits of the nurses from our cost calculation. The main goal of these visits was, apart from giving advice to patients, registration of health care consumption and outcomes, which is, of course, not common practice in normal daily care of patients.

Despite the high costs involved, detailed cost studies in the treatment of malignant disease and palliative therapy have received little attention. This may be due to the inherent difficulties in performing such studies. Follow-up of patients with malignant disease is sometimes difficult since the mortality rate is high, particularly among patients receiving palliative therapy (McQuay & Moore, 1994). In a palliative setting, it is sometimes difficult to differentiate between health care consumption, which can be attributed to the palliative stage of the disease or only to the treatment modality. If palliation-related costs were excluded, we found a decrease in the total costs of both treatments, but this did not affect the final conclusion that the total costs for brachytherapy and stent placement were similar.

This study focused on costs and not on efficiency. The primary aim of both treatments is to palliate symptoms rather than to improve the survival of oesophageal cancer. Both treatments resulted in an improvement of an important symptom of inoperable oesophageal cancer, that is, dysphagia. As survival of the two treatment groups was comparable, we did not perform a formal cost-effectiveness analysis. Despite a less rapid relief of dysphagia and a higher initial failure rate, brachytherapy was found to be an attractive alternative to stent placement in the palliation of malignant dysphagia, as brachytherapy was safer with fewer procedures needed for recurrent dysphagia (Homs *et al*, 2003b).

In conclusion, our study provides detailed insight into the total medical costs of two frequently used palliative treatments of dysphagia due to oesophageal cancer, that is, stent placement and brachytherapy. In spite of the higher initial costs for stent placement than for brachytherapy, total medical costs were similar. Therefore, cost considerations should not play an important role in decision making on the appropriate treatment strategy.

## Appendix A

The Dutch SIREC study group consisted of :

*Erasmus MC/University Medical Center Rotterdam:* Department of Gastroenterology & Hepatology: Marjolein YV Homs, Peter D Siersema, Ernst J Kuipers; Department of Public Health: Ewout W Steyerberg, Suzanne Polinder, Marie-Louise Essink-Bot; Department of Radiotherapy: Wilhelmina MH Eijkenboom; Department of Surgery: Hugo W Tilanus. *Academic Medical Center, Amsterdam:* Department of Radiotherapy: Lucas JA Stalpers; Department of Gastroenterology & Hepatology: Joep FWM Bartelsman; Department of Surgery: Jan JB van Lanschot. *University Medical Center Utrecht:* Department of Radiotherapy: Harm K Wijrdeman. *Rijnstate Hospital Arnhem:* Department of Gastroenterology: Chris JJ Mulder, Peter J Wahab. *Arnhem Radiotherapeutic Institute:* Janny G Reinders. *Antoni v Leeuwenhoek Hospital, Amsterdam:* Department of Gastroenterology: Henk Boot; Department of Radiotherapy Berthe MP Aleman. *Leyenburg Hospital, The Hague:* Department of Gastroenterology: Jan J Nicolai; Department of Radiotherapy: FM Gescher. *Medical Center Haaglanden, The Hague:* Department of Internal Medicine: Maarten AC Meijssen; Department of Radiotherapy: RGJ Wiggenraad. *Gelre Hospital, Apeldoorn:* Department of Internal Medicine: Jitty M Smit. *Reinier de Graaf Hospital, Delft:* Department of Gastroenterology: CJM Bolwerk.

Table A1

**Table A1:** Cost categories and data used in cost calculations

		**Data collection health care use**		
**Cost category**	**Parameter**	**CRF (physician)**	**Questionnaire (nurse)**	**Cost estimate (Unit price)**
*Treatment*				
Brachytherapy	Number	^*^		Real costs
Stent placement	Number	^*^		Real costs
				
*Inpatient days*				
Hospital (academic)	Days	^*^	^*^	Real costs
Hospital (general)	Days	^*^	^*^	Real costs
ICU	Days	^*^	^*^	Real costs
Nursing home	Days		^*^	Charges
				
*Health practitioner (inpatient)*				
Physician (academic)	Visits	^*^	^*^	Real costs
Physician (general)	Visits	^*^	^*^	Real costs
				
*Medical procedures*				
Endoscopy	Number	^*^		Real costs
PEG	Number	^*^		Charges
X-ray thorax	Number	^*^		Charges
X-ray stomach	Number	^*^		Charges
Ultra-sound scan stomach	Number	^*^		Charges
X-ray oesophagus	Number	^*^		Charges
ERT	Number	^*^		Charges
Other therapy	Number	^*^		Charges
				
*Extramural care*				
General practitioner (inpatient)	Visits		^*^	Fees
General practitioner (home visit)	Visits		^*^	Fees
Nursing care at home	Number		^*^	Charges
Nursing care at home (specialised)	Number		^*^	Charges
Drip-feed	Days		^*^	Charges

## References

[bib1] Adam A, Ellul J, Watkinson A, Tan BS, Morgan R, Sanunders M, Mason RC (1997) Palliation of inoperable oesophageal carcinoma: a prospective randomised trial of laser therapy and stent placement. Radiology 202: 344–348 9015054 10.1148/radiology.202.2.9015054

[bib2] Bartelsman JF, Bruno MJ, Jensema AJ, Haringsma J, Reeders JW, Tytgat GN (2000) Palliation of patients with esophagogastric neoplasms by insertion of a covered expandable modified Gianturco-Z endoprosthesis: experiences in 153 patients. Gastrointest Endosc 51: 134–138 10650253 10.1016/s0016-5107(00)70407-x

[bib3] Botterweck AA, Schouten LJ, Volovics A, Dorant E, van den Brandt PA (2000) Trends in incidence of adenocarcinoma of the esophagus and gastric cardia in ten European countries. Int J Epidemiol 29: 645–654 10922340 10.1093/ije/29.4.645

[bib4] Brewster AE, Davidson SE, Makin WP, Stout R, Burt PA. (1995) Intraluminal brachytherapy using the high dose rate microSelectrion in the palliation of carcinoma of the oesophagus. Clin Oncol (R Coll Radiol) 7: 102–105 7542470 10.1016/s0936-6555(05)80810-6

[bib5] Dallal HJ, Smith GD, Grieve DC, Ghosh S, Penman ID, Palmer KR (2001) A randomized trial of thermal ablative therapy versus expandable metal stents in the palliative treatment of patients with esophageal carcinoma. Gastrointest Endosc 54: 549–557 11677469 10.1067/mge.2001.118947

[bib6] Devesa SS, Blot WJ, Fraumeni Jr JF (1998) Changing patterns in the incidence of esophageal and gastric carcinoma in the United States. Cancer 83: 2049–2053 9827707

[bib7] Drummond MF, O’Brien B, Stoddard GL, GW T (1997) Methods for the Economic Evaluation of Health Care Programmes, 2nd edn. Oxford: Oxford University Press

[bib8] Farndon MA, Wayman J, Clague MB, Griffin SM (1998) Cost-effectiveness in the management of patients with oesophageal cancer. Br J Surg 85: 1394–1398 9782023 10.1046/j.1365-2168.1998.00916.x

[bib9] Gold MR, Siegel JE, Russel LB, Weinstein MC (1996) Cost-effectiveness in Health and Medicine. New York: Oxford University Press

[bib10] Homs MY, Eijkenboom WM, Coen VL, Haringsma J, van Blankenstein M, Kuipers EJ, Siersema PD (2003a) High dose rate brachytherapy for the palliation of malignant dysphagia. Radiother Oncol 55: 327–332 10.1016/s0167-8140(02)00410-3 12742273

[bib11] Homs MY, Steyerberg EW, Eijkeboom WM, Tilanus HW, Stalpers LA, Bartelmans JFWM, Lanschot JJB, Wijrdeman HK, Mulder CJJ, Reinders JG, Boot H, Aleman BMP, Kuipers EJ, Siersema PD for the Dutch SIREC Study Group (2003b) Single dose brachytherapy *versus* metal stent placement for the palliation of obstructive esophageal cancer: a randomized trial. submitted

[bib12] Homs MY, Steyerberg EW, Eijkenboom WM, Stalpers LJ, Bartelmans JFWM, Wijrdeman HK, Mulder CJJ, Reinders JG, Boot H, Aleman BMP, Siersema PD for the Dutch SIREC Study Group (2003c) Is high dose rate brachytherapy an alternative to stent placement in the palliation of malignant dysphagia – a randomized trial. Gastroenterology 124

[bib13] Jager J, Langendijk H, Pannebakker M, Rijken J, de Jong J (1995) A single session of intraluminal brachytherapy in palliation of oesophageal cancer. Radiother Oncol 37: 237–240 8746593 10.1016/0167-8140(95)01667-8

[bib14] Kozarek RA, Raltz S, Brugge WR, Schapiro RH, Waxman I, Boyce HW, Baillie J, Branch MS, Stevens PD, Lightdale CJ, Lehman GA, Benjamin S, Fleischer DE, Axelrad A, Kortan P, Marcon N, Branch S, Stevens P (1996) Prospective multicenter trial of esophageal Z-stent placement for malignant dysphagia and tracheoesophageal fistula. Gastrointest Endosc 44: 562–567 8934162 10.1016/s0016-5107(96)70009-3

[bib31] Lightdale CJ, Heier SK, Marcon NE, McCaughan Jr JS, Gerdes H, Overholt BF, Sivak Jr MV, Stiegmann GV, Rava HR (1995) Photodynamic therapy with porfiner sodium *versus* thermal ablation therapy with Nd:YAG laser for palliation of esophageal cancer: a multicenter randomized trial. Gastrointest Endosc 42: 507–512 8674919 10.1016/s0016-5107(95)70002-1

[bib15] Luketich JD, Christie NA, Buenaventura PO, Weigel TL, Keenan RJ, Nguyen NT (2000) Endoscopic photodynamic therapy for obstructing esophageal cancer: 77 cases over a 2-year period. Surg Endosc 14: 653–657 10948303 10.1007/s004640000144

[bib16] Lundell L, Leth R, Lind T Lonroth H, Sjovall M, Olbe L (1989) Palliative endoscopic dilatation in carcinoma of the esophagus and esophagogastric junction. Acta Chir Scand 155: 179–184 2472723

[bib17] McQuay H, Moore A (1994) Need for rigorous assessment of palliative care. BMJ 309: 1315–1316 7532500 10.1136/bmj.309.6965.1315 PMC2541847

[bib18] Moghissi K, Dixon K, Thorpe JA, Stringer M, Moore PJ (2000) The role of photodynamic therapy (PDT) in inoperable oesophageal cancer. Eur J Cardiothorac Surg 17: 95–100 10731642 10.1016/s1010-7940(99)00350-4

[bib19] Myrvold HE, Lundell L, Miettinen P, Pedersen SA, e.a. & Group t.N.G.S (2001) The cost of long term therapy for gastro-oesophageal reflux disease: a randomised trial comparing omeprazole and open antireflux surgery. Gut 49: 488–494 11559644 10.1136/gut.49.4.488 PMC1728480

[bib20] Nicholson DA, Haycox A, Kay CL, Rate A, Attwood S, Bancewicz J (1999) The cost effectiveness of metal oesophageal stenting in malignant disease compared with conventional therapy. Clin Radiol 54: 212–215 10210338 10.1016/s0009-9260(99)91153-4

[bib21] O’Donnell CA, Fullarton GM, Watt E, Lennon K, Murray GD, Moss JG (2002) Randomized clinical trial comparing self-expanding metallic stents with plastic endoprostheses in the palliation of oesophageal cancer. Br J Surg 89: 985–992 12153622 10.1046/j.1365-2168.2002.02152.x

[bib22] Pisani P, Parikin DM, Bray F, Ferlay J (1999) Estimates of the worldwide mortality from 25 cancers in 1990. Int J Cancer 83: 18–29 10449602 10.1002/(sici)1097-0215(19990924)83:1<18::aid-ijc5>3.0.co;2-m

[bib23] Sagar PM, Gauperaa T, Sue-Ling H, McNahon MJ, Johnston D (1994) An audit of the treatment of cancer of the esophagus. Gut 35: 941–945 7794305 10.1136/gut.35.7.941 PMC1374841

[bib24] Sahai AV (2000) Cost-effectiveness studies in endoscopy: are they worth it? Endoscopy 32: 986–990 11147950 10.1055/s-2000-9618

[bib25] Schraube P, Fritz P, Wannenmacher MF (1997) Combined endoluminal and external irradiation of inoperable oesophageal carcinoma. Radiother Oncol 44: 45–51 9288857 10.1016/s0167-8140(97)00083-2

[bib26] Siersema PD, Hop WC, Blankenstein Mea (2001) A conparison of 3 types of covered metal stents for the palliation of patients with dysphagia caused by esophagogastric carcinoma: a prospective, randomized study. Gastrointest Endosc 54: 145–153 11474382 10.1067/mge.2001.116879

[bib27] Siersema PD, Hop WC, Dees J, Tilanus HW, van Blankenstein M (1998) Coated self-expanding metal stents versus latex prostheses for esophagogastric cancer with special reference to prior radiation and chemotherapy: a controlled, prospective study. Gastrointest Endosc 47: 113–120 9512274 10.1016/s0016-5107(98)70342-6

[bib28] Spencer GM, Thorpe SM, Blackman GM (2002) Laser augmented by brachytherapy versus laser alone in the palliation of adenocarcinoma of the oesophagus and cardia: a randomised study. GUT 50: 224–227 11788564 10.1136/gut.50.2.224 PMC1773102

[bib29] Sur RK, Levin CV, Donde B, Sharma V, Miszczyk L, Nag S (2002) Prospective randomized trial of HDR brachytherapy as a sole modality in palliation of advanced esophageal carcinoma – an International Atomic Energy Agency study. Int J Radiat Oncol Biol Phys 53: 127–133 12007950 10.1016/s0360-3016(02)02702-5

[bib30] Taal BG, Aleman BM, Koning CC, Boot H (1996) High dose rate brachytherapy before external beam irradiation in inoperable oesophageal cancer. Br J Cancer 74: 1452–1457 8912544 10.1038/bjc.1996.564 PMC2074787

